# Use of Antimony in Ophthalmia

**Published:** 1813-06

**Authors:** George Fielding

**Affiliations:** Hull


*To the Editors of the Medical and Physical Journal.*

GENTLEMEN,

IN the last Number of your very useful Journal, there is a valuable communication by Mr. Adams, on the use of Emetic Tartar in the Egyptian Ophthalmia, and from much experience I am persuaded that the efficacy of that remedy is not overrated by him.

In confirmation of what Mr. Adams has advanced, I beg to observe, that I have used the same medicine in nauseating doses for the purpose of allaying inflammation in different diseases of the eyes, ever since the year 1807, both in private practice and in the Infirmary here. I was first led to the use of this remedy by reading two very curious cases related by Mr. Wardrop, in his publication on diseases of the eyes. These cases are so valuable and important, that they can scarcely have escaped the recollection of those who have read Mr. Wardrop's book. In both these instances the patients had been blind a long time, from opacity of the cornea, and both are stated to have recovered so much a short time before death, as to have had the pleasure of seeing their relatives again. I have not the publication by me at present, but, to the best of my recollection, either one or both of the patients died of pulmonary consumption.

It

It immediately occurred to me, that if, in imitation of this natural process, a powerful and long-continued action of the absorbents could be artificially produced, some benefit might possibly be derived in similar cases. The emetic tartar seemed the most likely to effect this purpose, given in such a manner as to keep up frequent long-continued nausea. I have accordingly used this remedy in opacity of the cornea ever since, and with very great success; and, though sometimes other means have been conjoined along with the antimony, such as occasional doses of calomel, the division of turgid vessels, and the application of solutions of oxymuriat hydrargyri to the eye itself, yet my principal dependance has been upon the antimony.

Seeing the good effects in this disease, I extended the use of the remedy to those cases of inflammation which sometimes succeed to couching, and other operations on the eyes, and afterwards to cases of ophthalmia from other causes, with effects which far exceeded my expectations. To have the full benefit of this or perhaps any other remedy in the Egyptian or any other acute species of ophthalmia, it is of importance that it should be applied very early, otherwise such is the rapid progress of the disease, that the organ will often be permanently injured; but even after the complaint has existed some days, its further progress may be arrested, but with more difficulty than in the incipient stage. I have only seen one instance of Egyptian ophthalmia (six weeks ago) in which the antimony after two days trial appeared to have been of no service. Large bleedings, and occasional purgatives were then joined with it, but the disease continued its progress. At the end of the week the aqueous humor was discharged by puncturing the cornea, as recommended by Mr. Wardrop; the pain was almost immediately relieved, the progress of the inflammation was stopped, and the patient recovered perfect vision in less than a fortnight afterwards. In scrofulous ophthalmia it is, in my opinion, the most efficacious general medicine which has hitherto been employed; but here again its best effects will be seen at the commencement of the complaint; and if given at the very beginning of two or three succeeding attacks, my experience in a great number of cases of this nature enables me to say, that it will generally destroy the propensity to return altogether.

In the purulent ophthalmia of infants I have not found it necessary to use the antimony, and have felt a reluctance, on account of its severity; but there is no doubt that there are occasionally cases of this nature in which it may be necessary, and that it would be extremely useful. In the common and in in the Egyptian ophthalmia, and whenever inflammatory symptoms succeed to operations on the eyes, the antimony will often supersede the lancet altogether; and when this does not happen, bleeding, according to the urgency of the symptoms, will be rendered much more effectual than it otherwise would be by being employed in conjunction with the antimony. I am, Gentlemen,

Your obedient Servant,

*Hull, April* 10, 1813. GEORGE FIELDING.

